# A Study of Anemia Among Adolescent Females in the Urban Area of Nagpur

**DOI:** 10.4103/0970-0218.43230

**Published:** 2008-10

**Authors:** Sanjeev M Chaudhary, Vasant R Dhage

**Affiliations:** Department of Preventive and Social Medicine, Government Medical College and Hospital, Nagpur, Maharashtra, India

**Keywords:** Adolescent female, anemia, height, urban, weight

## Abstract

**Objectives:**

To estimate the prevalence of anemia among adolescent females and to study the socio-demographic factors associated with anemia.

**Materials and Methods:**

A cross-sectional survey was conducted in an urban area under Urban Health Training Center, Department of Preventive and Social Medicine, Government Medical College and Hospital, Nagpur. A total of 296 adolescent females (10–19 years old) were included in this study. The study took place from October 2002 to March 2003 (6 months). Statistical analyses were done using percentage, standard error of proportion, Chi-square test, and Student‘s ‘t’ test.

**Results:**

The prevalence of anemia was found to be 35.1%. A significant association of anemia was found with socio-economic status and literacy status of parents. Mean height and weight of subjects with anemia was significantly less than subjects without anemia.

**Conclusions:**

A high prevalence of anemia among adolescent females was found, which was higher in the lower socio-economic strata and among those whose parents were less educated. It was seen that anemia affects the overall nutritional status of adolescent females.

## Introduction

Adolescence has been defined by the World Health Organization as the period of life spanning the ages between 10 to 19 years.(1) This is the formative period of life when the maximum amount of physical, psychological, and behavioral changes take place. This is a vulnerable period in the human life cycle for the development of nutritional anemia, which has been constantly neglected by public health programs. Girls are more likely to be a victim due to various reasons. In a family with limited resources, the female child is more likely to be neglected. She is deprived of good food and education, and is utilized as an extra working hand to carry out the household chores. The added burden of menstrual blood loss, normal or abnormal, precipitates the crises too often. This study was planned to highlight the problem of anemia in adolescent females and to study socio-demographic factors related to anemia.

## Materials and Methods

The Urban Health and Training Center in Bapunagar is an urban field practice area attached to the Department of Preventive and Social Medicine, Government Medical College and Hospital, Nagpur. The present study was conducted at Bhande Plot Area. This area was selected by a simple random sampling from among the beneficiary areas under this center. The population of this area is about 4,000. Initially, a pilot study was conducted to pretest the proforma and to have a rough estimate of the prevalence of anemia. The prevalence was found to be 34% in this pilot study. Taking *P* = 0.34, allowable error (d) = 10% of *P*, and using this in the formula of sample size *n* = Z^2^_1- α_ *P* (1-*P*) /d^2^, sample size(2) was estimated to be 176.

To have an effective coverage, it was decided to cover the whole area. The study was approved by the ethical committee of the Government Medical College and Hospital in Nagpur.

A house-to-house survey was carried out by the investigator. After obtaining written informed consent from the head of the household, information about the socio-demographic characteristics was recorded in the predesigned, pretested proforma. This was followed by a clinical examination of the subject including height and weight. Socio-economic status (SES) was estimated according to a modified Kuppuswamy's scale. The total number of members in the family constituted the ‘family size’. For hemoglobin estimation, 20 μl of capillary blood was taken in a hemoglobinometer pipette and transferred to a prenumbered glass bottle containing 5 ml Drabkins reagent. Hemoglobin estimation was done by the cyanmethaemoglobin method(3) using a Klett-Summerson photoelectric colorimeter with green filter (500–570 nm wave length). For typing of anemia, a thin blood smear was prepared on a prenumbered clean glass slide and allowed to dry. Smears were stained with Leishman's stain and examined under the high power of the microscope. An expert opinion of a pathologist was sought while examining the slides. The next day, the results of the hematological investigations were conveyed to the subjects and those found to have anemia were given appropriate treatment and advice regarding proper diet.

### Criteria for anemia

Hb < 12 gm% for nonpregnant adolescent, Hb < 11 gm% for pregnant adolescent.(4)

### Statistical analyses

The statistical analyses were done using Chi-square test, Students t test, mean, standard error of proportion, etc. The help of a statistician was sought while analyzing the data.

## Results

Out of 296 subjects, 104 (35.1%) subjects were found to be anemic. A statistically significant association of anemia was found with the socio-economic status of study subjects, though this association may not hold true for higher socio-economic status due to a smaller sample size in that group. None of the subjects belonged to socio-economic strata I (upper) and V (lower). A statistically highly significant association of anemia was found with the mother‘s and father’s educational status. Other factors like age group, attainment of menarche, type of family, family size, and type of diet were not significantly associated with anemia [Table 1].

**Table 1:** Socio-demographic correlates of anemia in adolescent females

Factor	No. of subjects (n = 296)	Subjects with anemia (n = 104)		χ^2^	Degrees of freedom	p
					
		No.	%(Standard error)			
SES						
II (upper middle)	64	21	32.8 (5.86)			
III (lower middle)	116	32	27.6 (4.15)			
IV (upper lower)	116	51	43.9 (4.60)	6.96	2	0.029
Mothers education^a^						
Illiterate	13	10	76.9 (11.68)			
Primary	80	40	50.0 (5.59)			
Middle	72	20	27.8 (5.27)			
SSC and above	124	33	26.6 (3.96)	22.9	3	0.000
Fathers education^b^						
Illiterate	21	15	71.4 (9.86)			
Primary	24	14	58.3 (10.06)			
Middle	28	14	50.0 (9.44)			
SSC and above	201	52	25.8 (3.12)	28.0	3	0.000

a Mother of 7 subjects not alive, hence excluded.

b Father of 22 subjects not alive, hence excluded

### Severity of anemia

Out of 104 subjects, 72 subjects (69.2%) had mild anemia [Hb 10 to < 12 gm%] while 32 subjects (30.8%) had moderate anemia [Hb 7 to < 10 gm%]. None of the subjects had severe anemia.(5)

There were 4 married subjects of whom 2 were pregnant. One of them had mild anemia [Hb 10 to < 11 gm%].

It was found that mean height and mean weight of subjects with anemia is less as compared with that of subjects without anemia; the difference was statistically significant [Table 2].

**Table 2:** Comparison of mean height and mean weight of subjects with and without anemia

Variable	Subjects with anemia (n=104)	Subjects without anemia (n=192)	t, df, p
Mean height (cm)	142.2 (SD = 10.2)	144.6 (SD = 9.2)	2.06, 294, 0.02
Mean weight^a^(kg)	31.2(SD = 7.7)	33.5(SD = 7.2)	2.83, 292, 0.02

SD = standard deviation.

a Pregnant subjects excluded. df = Degrees of freedom

It was found that 15.9% of subjects had early stages of iron deficiency reflected by normocytic hypochromic picture. A total of 25.4% subjects had iron deficiency anemia while 4.7% of subjects had dimorphic anemia [Table 3].

**Table 3:** Peripheral smear examination

Red cell morphology	Subjects with anemia (n = 104)	Subjects without anemia (n = 192)
Normocytic normochromic	00	160
Normocytic hypochromic	15	32
Microcytic hypochromic	75	00
Dimorphic	14	00

## Discussion

The overall prevalence of anemia was found to be 35.1%. Similar prevalence is reported by CMS Rawat *et al.*(6) at Meerut. A higher prevalence was noted by J Rajaratnam *et al.*(7) in Tamil Nadu. Toteja GS *et al.*(8) found 90.1% prevalence of anemia among adolescent girls from 16 districts of India, with 7.1% having severe anemia. In this study, a significant association of anemia was found with socio-economic status, which may be due to the availability of high quality food with better socio-economic status. A significant association of the prevalence of anemia with educational status of parents reflects better awareness among literate mothers, as well as better socio-economic conditions. None of the subjects had severe anemia. Bulliyy *et al.*(9) found 96.5% prevalence among non school going adolescent girls in three districts of Orissa, of which, 45.2%, 46.9%, and 4.4% had mild, moderate, and severe anemia. They found significant association between Hb concentration and the educational level of girls, their parents' family income, and body mass index. In the present study, mean height and mean weight of subjects with anemia was significantly less than subjects without anemia, which suggests that anemia affects the overall growth of adolescents. The majority of subjects with anemia in the present study (25.4%) had a microcytic hypochromic picture in the peripheral smear suggestive of iron deficiency anemia, while 4.7% of subjects had a dimorphic picture. Khanduri *et al.*(10) found peak incidences of megaloblastic anemia in the age group of 10–30 year olds (48%) with female preponderance (71%) in India.

## Conclusions and Recommendations

The overall prevalence of anemia among adolescent females was found to be 35.1%. It is seen that anemia affects the overall nutritional status of adolescent females. A significant association of anemia with socio-economic status and parents' educational status suggests a need to develop strategies for intensive adult education and to improve the socio-economic status of the population through poverty alleviation programs. This should be supported by programs for the prevention of anemia among adolescent girls through nutrition education and anemia prophylaxis.
